# Application of Big Data Analysis on the Relationship between Career Delayed Gratification and Organizational Socialization Outcomes for New Generation Employees

**DOI:** 10.1155/2022/6065435

**Published:** 2022-07-19

**Authors:** Gaoxi Hu, Ting Nie, Tenfeng Qiu, Guifeng Tian, Yaozhong Liu

**Affiliations:** ^1^Guangzhou Institute of Science and Technology, Zhongkai University of Agriculture and Engineering, Guangzhou 510023, Guangdong, China; ^2^School of Business, Macau University of Science and Technology, Macau 999078, China; ^3^School of Public Administration, Guangdong University of Finance, Guangzhou 510521, Guangdong, China; ^4^Guangdong Construction Polytechnic, Guangzhou 510440, Guangdong, China; ^5^Guangzhou Huashang College, Guangzhou 511399, Guangdong, China

## Abstract

The effect of occupational delayed gratification is that it can prompt individuals to postpone short-term instant gratification in order to pursue more valuable long-term career goals when they encounter a choice of interests, which is very beneficial to the career development of the new generation of employees. Based on extensive literature review and interviews, this paper compiles a pretest questionnaire for investigation and proposes models and hypotheses. For the data of the pretest questionnaire, big data item discrimination analysis, Cronbach's alpha internal consistency reliability test, and exploratory factor analysis were used to eliminate unreasonable items. From the four aspects of self-evaluation, self-efficacy, self-esteem, and social support, it explains how to cultivate and improve the career delayed gratification of the new generation of employees, so that they can make the right choice between short-term and long-term benefits, setbacks, and success, thereby improving the organizational socialization outcome relationship.

## 1. Introduction

With the advent of the new economic era dominated by knowledge and information, the global financial crisis has intensified, and knowledge capital has gradually replaced financial capital and has risen to the status of strategic resources. Enterprises pay more attention to human capital investment and long-term returns. Therefore, humanity management in enterprise management is more and more dominant [1–3]. As the core of human capital investment, career management has become an important tool to achieve a win-win situation for enterprises and individuals coordinated development. Modern enterprises must also expand from the incentive mechanism centered on interests to the dual management of people-oriented and high satisfaction. As an important part of self-career management, occupational delayed gratification occupies a major position in enterprise management. Therefore, in order to occupy a seat in the fiercely competitive market, enterprises must not only compete for talents, but more importantly, strive to make organizations and talents develop together [4–6].

Undoubtedly, we should not only focus on satisfying the current development, but also need to look to the future and consider the long-term development goals of higher value and sustainable development. Therefore, occupational delayed gratification is particularly important from both an organizational and an individual perspective. Therefore, fundamentally understanding the psychological quality and career maturity of employees plays an important role in the effective management of enterprise employees.

At present, post-80s employees enter the workplace and gradually become the backbone of enterprises. Most of them are well-educated and knowledge-based employees. The value they create for enterprises in terms of productivity and creativity is immeasurable [7–10]. Knowledge-based talents are a group who are particularly focused on their career growth and job achievement, and they attach great importance to their career development. However, the post-80s employees pay more attention to personal feelings and subjective perceptions in their work, with strong subjective will, weak self-selection ability, and lack of a clear sense of belonging. In addition to the endless temptations in the current Internet age, work pressure is gradually increasing. The turnover rate is also increasing, which poses new challenges to the management of enterprises [11–13]. How to effectively improve employees' satisfaction and loyalty to the enterprise, stimulate employees' work enthusiasm and initiative, cultivate and retain talents, and finally achieve a win-win situation for the enterprise and employees is one of the problems that enterprises urgently need to solve at present, a topic worthy of study and discussion.

This paper has carried out in-depth research on the occupational delayed gratification of knowledge-based talents, which has certain theoretical and practical significance. For individuals, through appropriate occupational delayed gratification, employees can be guided by more valuable long-term goals in the future in their career choices or work, and at the same time, it can also help the continuous development of personal comprehensive ability, so that the ultimate goal can be achieved. The possibility is greatly improved. For an organization, hiring employees with a higher level of occupational delayed gratification is conducive to achieving common goals between the organization and employees, improving the loyalty and core competitiveness of employees, and promoting the organization's future sustainable and high-speed development.

## 2. Theoretical Background

### 2.1. Dimensions of Organizational Socialization

Fisher (1986) proposed a four-dimensional structure of organizational socialization through empirical research [14]. He believed that the content of organizational socialization includes four aspects: the first aspect is to learn organizational-related knowledge. Fisher believes that there are many aspects of the organization that new employees need to learn, some of which are explicit, such as the specified work standards, reward and punishment standards, and personnel promotion standards; some are implicit, such as employees attitudes towards work, personal beliefs and work values, interpersonal expressions, and corporate culture. The second aspect is learning how to function in group collaboration. Similar to the previous description, what new employees need to learn after entering a new organization or team is also divided into two categories: explicit and implicit. The explicit content includes the names of members, the responsibilities and obligations to be performed in the work, and teamwork and communication skills; the implicit content includes the group's hidden standards and codes of conduct, and team culture. The third aspect is the study of work-related knowledge. The fourth area is personal learning and improvement. Organizational socialization can prompt individuals to face up to their true professional skills, work potential, and deficiencies.

**Figure 1:**
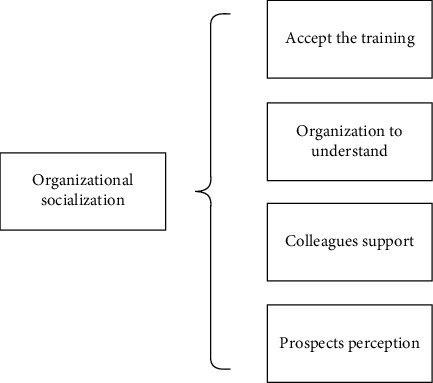
The organizational social structure.

Since then, Ostroff and Kozlowski in 1992 also further integrated the social content dimension of employee organization into four parts [15]: task, role, group, and organization. This is very close to Fisher's four-dimensional structure theory. Chao et al. conducted an empirical study on 594 American college graduates through a large number of literature review and analysis and used factor analysis to explore the content dimension of organizational socialization [16]. Chao et al. concluded that the content structure of employee organizational socialization covers six dimensions: job performance standardization, interpersonal relationships, organizational politics, language, organizational goals/values, and history. Taormina (1997) limited the research object of organizational socialization to employees in mainland China, Hong Kong, and Singapore. Through empirical research, the organizational socialization content dimension is constructed into four dimensions [17], which are the degree of training, the degree of organizational understanding, the degree of support from colleagues, and the expectations for the future. The four-dimensional theory proposed by Taormina et al. is more universal in the world, so it is widely accepted by scholars. Thomas and Anderson (1998) also agreed that organizational socialization is composed of four dimensions. Based on Taormina's research, they further proposed that the components of organizational socialization are role orientation, social environment, interpersonal resources, and organizational knowledge. The organizational social structure is shown in Figure 1.

### 2.2. Organizational Socialization Strategy and Employee Active Socialization Behavior

The organization's socialization strategy mainly refers to the organization as the main body of action, in order to assist new employees to integrate into the organization, speed up the process of adapting to the organizational environment, increase employees' sense of job security, reduce anxiety, and understand and master the necessary skills as members of the organization, methods of work attitude, and code of conduct. In the early research on organizational socialization by foreign scholars, the process of employee organizational socialization is usually regarded as the process of employees passively adapting to the new organizational environment, so the socialization strategy adopted by the organization plays a major role in this process. Even the research in this field has developed to the present, and the organizational socialization strategy is also one of the focuses in this research field. In recent domestic and foreign scholars' research on organizational socialization, experts and managers have further concluded the following.

The organizational socialization process of employees is not completely passive; on the contrary, new employees also assume an important position in the process of entering the organization. They actively adapt to organizational norms and culture by learning organizational culture, understanding organizational information, and building good relationship networks with other members of the organization.

Louis (1980) regarded this active socialization behavior of employees as the behavior of new employees actively understanding organizational information in the process of adjusting their own positioning and changing roles, trying to improve their speed of adapting to the organizational environment, and mastering the work content. On this basis, Mitchell believes that the active socialization behaviors that employees take are generated by actively seeking opportunities for interaction between organizational and team members. The content of the behavior mainly covers consultation between members of the organization, talking in other members' offices or work areas, and joining parties and other social activities.

### 2.3. Measures of Organizational Socialization

For the measurement and evaluation criteria of organizational socialization strategies, the Organizational Socialization Strategy Scale, which integrates six strategies of organizational socialization, compiled by Jones (1986) has generally been supported by scholars. The scale integrates six representative strategies of organizational socialization, and each strategy contains five items, covering a total of thirty items.

Cable and Parsons (2001) improved and revised the Organizational Socialization Strategy Scale developed by Jones on the basis of empirical evidence. In 2005, Kim et al. agreed with the above point of view and used the revised scale in a subsequent empirical study with Korean employees as the subject sample. The study confirmed that the scale revised by Cable et al. has high measurement reliability. They constructed the other twenty-six items as a set (Ashforth et al., 1995), and the higher and lower mean of the total score of the whole set confirms the institutional degree of the organization's adoption of socialization strategies and can be achieved by providing systematic and sophisticated activities for the organization, reducing insecurity awareness among new recruits. The focus of the employee active social behavior measurement questionnaire developed by Ashford and Black includes collecting information (including four items, reliability 0.78), seeking feedback (including four items, reliability 0.92), socialization (including three items, 0.84)building a relationship network (three items, reliability 0.82), evaluation of superior-subordinate relationship (three items, reliability 0.82), work communication and work adjustment (three items, reliability 0.90), and so on.

## 3. Big Data Analysis Organization Socialization

### 3.1. Questionnaire Design

In order to improve the survey questionnaire and try to control the scientificity and accuracy of the data, we selected three different enterprises in Dalian to conduct a small-scale survey on the basis of preliminary qualitative analysis and purification of the items. The questionnaire items were adjusted or deleted through statistical analysis of small-scale sample data, which further improved the setting of the questionnaire items and formed a formal questionnaire and applied it to the later large-scale data research.

The formal questionnaire is divided into four parts: the first part, the basic information of the subjects, including gender, age, education, job type, position level, and company nature. The second part is the entry expectation part, which includes 4 items of job expectations, 4 items of team expectations, and 3 items of enterprise expectations, for a total of 11 items. The third part, psychological capital, includes 6 items of optimism, 4 items of hope, 4 items of self-efficacy and 2 items of resilience, a total of 16 items; the fourth part, organizational socialization, includes received training on 7 items, organization to understand 6 items, colleagues support 4 items, and prospect perception 4 items, a total of 21 items.

### 3.2. Statistical Analysis

The survey method adopts the form of questionnaire to collect data. A total of 600 paper questionnaires were distributed in the formal survey, 432 valid questionnaires were recovered, and the recovery rate of valid questionnaires was about 72%. At the same time, 300 electronic questionnaires were distributed, 160 valid questionnaires were recovered, and a total of 592 valid questionnaires were recovered.

In this survey, male employees surveyed accounted for 55.07% of the total number of employees, and female employees accounted for 44.93%, and the ratio of male and female employees was basically the same. Since this survey is not conducted for a certain gender group, and ideally the number of male and female employees accounts for half of the total number of employees, this gender distribution is basically consistent with the preconceived idea.

**Figure 2:**
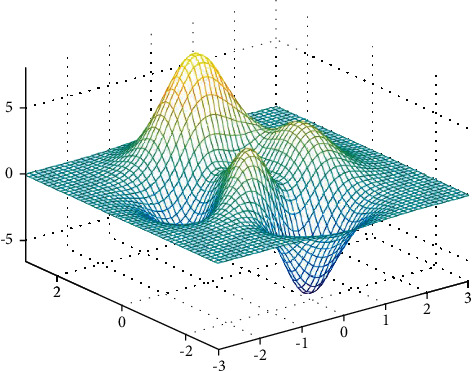
Correlation of factors.

The majority of those surveyed were under the age of 30, accounting for 98.82% of all respondents, and 31–35. The age-old subjects accounted for 1.18% of the total number of subjects, which showed that most of the subjects in the study were young people. Most of the surveyed sample groups are college graduates or college graduates, accounting for 87.5% of the respondents, and 3.72% of the graduates. In Figure 2, the points determined by the abscissa and ordinate represent the correlation between different variables.

This survey mainly involves state-owned enterprises, foreign-funded enterprises, Sino-foreign joint ventures, and private enterprises. Among them, the number of private enterprises accounted for 46.79% of the total number, ranking first. Sino-foreign joint ventures and state-owned enterprises followed, accounting for 25.84% and 16.39% of the total sample, respectively.

Among the industries involved in the sample group of this survey, the number of people in the service industry is the majority, accounting for 43.58%; the number of people in manufacturing ranked second, accounting for 31.08%.

Most of the job types are technical personnel, accounting for 63.68% of the total sample population, and the proportions of functional personnel and marketing personnel are similar.

Since the selected samples belong to new employees, grass-roots employees account for the vast majority in terms of position level, reaching 81.76%, and 13.85% of the subjects are middle-level employees, and no samples of senior employees have been obtained.

### 3.3. Reliability Analysis of Big Data

The Organizational Socialization Scale in the large sample test has a total of 21 items. We conducted reliability analysis on each item of the training organization's understanding of colleague support and prospect prediction and evaluated all the questions under the four dimensions of organizational socialization. The reliability analysis was carried out, and the results are shown in Table 1. In Table 1, the Cronbach's coefficient of the organizational socialization measurement scale is 0.947, and the Cronbach's coefficient of the training dimension is 0.946, and the Cronbach's coefficient of the organizational understanding dimension is 0.951. The Cronbach's coefficient of the colleagues support dimension is 0.872, and the Cronbach's coefficient of the prospect prediction dimension is 0.855, both above 0.7, indicating that the questionnaire has good reliability.

### 3.4. Correlation Analysis Based on Big Data

In the formal large sample test, Pearson's correlation coefficient was used for correlation analysis. Choose a two-tailed test to judge the correlation between variables. The correlation coefficient between the variables is positive or negative. The correlation coefficient is greater than 0, indicating that there is a positive correlation between the variables, and the correlation coefficient is less than 0, indicating that there is a negative correlation between the variables. The correlation coefficients between variables are different, indicating that the degree of correlation between variables is different. The correlation coefficient analysis is shown in Table 2.

**Figure 3:**
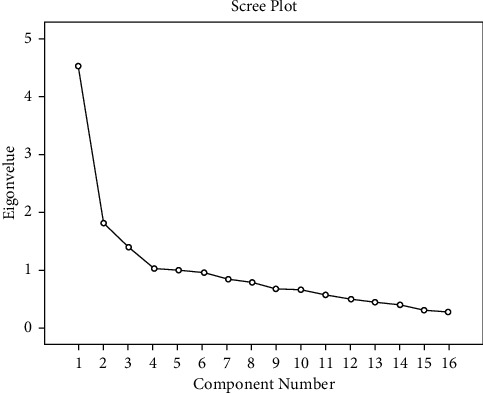
The factors meet the requirements.

The experiment tested the remaining 16 measurements, variables Kaiser–Meyer–Olkin Measure of Sampling Adequacy value of 0.865, greater than 0.8, suitable for factor analysis, and the Bartlett Sphericity test X2 is 465.408 (55 degrees of freedom, *P* < 0.001), which is significant, indicating that there are common factors among correlation matrices of the parent population, which is suitable for factor analysis on this basis. Above, the principal component factor analysis method is adopted to extract factors. As shown in Figure 3, 16 factors meet the requirements.

## 4. Occupational Delayed Gratification Optimizes Organizational Social Relationships

The effect of occupational delayed gratification is that it can prompt individuals to postpone short-term instant gratification in order to pursue more valuable long-term career goals when they encounter a choice of interests, which is very beneficial to the career development of employees. This section describes how to cultivate and improve employees' career delayed gratification from four aspects: self-evaluation, self-efficacy, self-esteem, and social support, so that employees can make the right choice between short-term and long-term benefits, setbacks, and success, thereby improving the entrepreneurial energy efficiency of employees.

### 4.1. Cultivating Employees to Have a Positive Core Self-Evaluation

Core self-evaluation is the corresponding evaluation and conclusion of self-worth given by an individual at the subconscious level. Positive core self-evaluation often focuses on the attention and discovery of one's own strengths. Most individuals who are accustomed to positive self-evaluation will face setbacks positively. To cultivate employees' positive core self-evaluation, it should be done from all aspects of education and teaching.

Expectation effect tells us that when we give more positive attention to the individual, give timely positive feedback to his achievements, and always face him with upward and positive force, the individual will perceive our expectations and will move towards him. We work hard in the desired direction and finally achieve self-realization. In order to improve the core self-evaluation power of employees in the process of starting a business in the future, the society should make good use of the expectation effect, actively encourage employees, and let them perceive the positive energy. Employees use this power to guide their own lives in the future.

In order to enable employees to internalize positive core self-evaluation into their own introspective power, they should be appropriately targeted when organizing employee activities, fully consider the existing psychological characteristics and growth characteristics of employees, and organize some activities that are more engaging and interesting, such as carrying out psychological games and trusting seats back to back, so that employees feel trust in the activities. The strength of persistence and the cultivation and improvement of willpower carried out through such activities, on the one hand, mobilize the enthusiasm of employees to participate, and on the other hand, they also convey that the positive energy of trust and persistence develops employees' potential in solidarity and cooperation, improves employees' self-confidence, and thus enhances employees' core self-evaluation.

At present, the combination of work and study is an important talent training mode in various technical colleges and universities, and its benefits in all aspects have been demonstrated and recognized by many parties. For the goal of improving the energy efficiency of entrepreneurship for employees, make full use of the factory environment and consciously cultivate employees to bear hardships and stand hard work. The willpower and ability to deal with problems are also issues that need to be paid attention to by technical colleges today. Among the employees who are about to enter the working environment, a considerable number of them grew up in a superior and coddled environment and cannot well adapt to the company's requirements for their work intensity and efficiency. Faced with a solution, they often choose to withdraw and give up, which is obviously not conducive to the cultivation of their entrepreneurial ability. Therefore, relevant enterprises should do the corresponding publicity work in advance, so that employees have a preliminary understanding of the formal process to be experienced and reduce the psychological gap of employees.

### 4.2. Improving Employee Self-Efficacy

Studies have found that individuals with higher levels of self-efficacy generally have a stronger motivation for achievement and are willing to put in more effort and wait to achieve their goals. Individuals with strong achievement motivation tend to be more active when encountering difficulties and setbacks, and they are less likely to take adverse coping methods such as avoidance and withdrawal. Pay attention to cultivating the self-confidence of employees, such as grasping the bright spots of employees, giving them more affirmative evaluation and encouragement, and carrying out community activities to create opportunities for employees to show themselves in various ways; through clever design, let employees learn in the process and experience the joy of success, etc. Improving the self-confidence of employees, stimulating and promoting their formation of high achievement motivation, forming their own sense of responsibility for the future, and having enough confidence to support their actions will ultimately improve employees' self-efficacy.

### 4.3. Improving Employee Self-Esteem

Individuals with strong self-esteem are more willing to give themselves stronger support in self-action, thereby enhancing self-action. For employees who start a business, everything in the future is new and unfamiliar, and they need to mobilize all their strength and determination to face various difficulties encountered in the process of starting a business. Individuals with low self-esteem tend to give low self-evaluation, easily feel the oppression of difficulties before taking action, and reduce their efforts to start a business. Enterprises can start from the three pillars that constitute self-esteem (self-love, self-confidence, and self-view) and improve employees' self-esteem through caring and respecting employees, communication and trust, and positive guidance, example learning, and action training.

### 4.4. Helping Employees Learn to Use and Feel Social Support

Humans are creatures of social life, and people grow up with mutual support and help. For entrepreneurial employees, the power of social support cannot be ignored. Social support refers to both the material support given to the individual and the emotional support given to the individual. The existence of these supports enables individuals to obtain both external and psychological satisfaction, which is beneficial for individuals to improve occupational delayed satisfaction in the process of entrepreneurship and ultimately obtain higher entrepreneurial energy efficiency.

Improving employees' sense of social support can start with employees' interpersonal communication and perception. First of all, it is necessary to help employees establish the awareness of active communication and then train their communication behaviors and improve their communication skills by organizing life-like activities. In addition, to improve the perception of employees, when there is social support but the employees do not feel it, the support effect will be greatly reduced, so it is very necessary to cultivate the perception of employees. Through this kind of learning and training, employees can see their social support resources at a glance; they can always maintain the sensitivity of perception and utilization of social support forces, better improve their ability to withstand setbacks in entrepreneurship, and in the future work and entrepreneurship can also achieve a multiplier effect.

In a word, in the process of cultivating entrepreneurial employees, teachers should grasp all aspects that affect employees' entrepreneurial ability, start with improving their self-confidence and self-esteem, and ensure that employees have a positive core self-evaluation ability, so that they can better manage and utilize their own social support resources, improve employees' career delayed gratification, enable employees to make the right choice between short-term and long-term benefits, setbacks, and success, obtain high entrepreneurial energy efficiency, and lay a solid foundation for their future entrepreneurial development creating condition.

## 5. Conclusion

Based on the literature review and discussion on entry expectations, psychological capital, and organizational socialization, this paper proposes a hypothetical study on the relationship between entry expectations, psychological capital, and organizational socialization and collects data through questionnaires for empirical analysis to obtain entry expectations and analysis results of three dimensions and four dimensions of psychological capital and organizational socialization

The limitations of this paper are mainly shown as follows: First, the samples selected are all new employees who joined the company within two years. In order to ensure the diversity of samples, employees from different industries are selected as subjects as possible. However, due to the limitations of research conditions, the number of people in such industry distribution is not balanced, so the representativeness of research conclusions is limited. Secondly, some values of employees' psychological capital and organizational socialization support dimensions did not get the expected effect. The results of the questionnaire were limited by the fact that the researchers did not adequately monitor the actual situation of the employees filling out the questionnaire data, the limitations of traditional Chinese thinking, and the weak ability of the employees to withstand the environment. There are still some psychological barriers. Therefore, the data obtained from a few questionnaires issued by the company's leaders may be of limited representativeness. Thirdly, the new employee onboarding expectation in this paper is regarded as a value at a static point, and the development cycle of the onboarding expectation level and the degree of development and change in different time series are not studied.

In the future research, we plan to study the new employee management of a specified industry or enterprise type to make the research more pertinent. In addition, the same batch of samples is to be selected for data tracking, to study the interaction effect of adjusted entry expectations and organizational socialization, as well as the moderating effect of psychological capital on the expectation gap.

## Figures and Tables

**Table 1:** Reliability tables for organizational socialization tables.

Name of scale	Dimension	Deleted value	Cronbach's alpha	Standardized Cronbach's alpha
Accepting the training	OP1	0.943	0.946	0.947
	OP2	0.941		
	OP3	0.941		
Organization to understand	HO1	0.941	0.951	
	HO2	0.940		
Colleagues support	SE1	0.942	0.872	
	SE2	0.944		
	SE3	0.944		
	SE4	0.942		
Outlook	RE1	0.941	0.855	
	RE2	0.942		
	RE3	0.943		

**Table 2:** Pearson correlation coefficient matrix of onboarding expectation, psychological capital, and organizational socialization variables.

	Organization to understand	Colleagues support	Prospects perception	Optimistic	Self-efficacy	Companiesexpect
Organization to understand	1	0.747	0.803	0.561	0.675	0.509
Colleagues support	0.747	1	0.849	0.721	0.726	0.632
Prospects perception	0.803	0.849	1	0.478	0.751	0.632
Optimistic	0.561	0.721	0.478	1	0.690	0.989
Self-efficacy	0.675	0.726	0.751	0.690	1	0.815
Companies expect	0.509	0.508	0.632	0.989	0.815	1

## Data Availability

The raw data supporting the conclusions of this article will be made available by the corresponding author, without undue reservation.
